# Fetal and postnatal metal metabolism–related changes in brain function are associated with childhood behavioral deficits

**DOI:** 10.1126/sciadv.adz1340

**Published:** 2026-04-24

**Authors:** Elza Rechtman, Avraham Reichenberg, Azzurra Invernizzi, Lazar Fleysher, Vida Rebello, Kristie Oluyemi, Michelle A. Rodriguez, Anna Sather, Libni A. Torres-Olascoaga, Luis F. Bautista-Arredondo, Sandra Martínez-Medina, Rafael Lara-Estrada, Chris Gennings, Martha M Téllez-Rojo, Robert O. Wright, Manish Arora, Megan K. Horton

**Affiliations:** ^1^ Department of Environmental Medicine and Climate Science, Icahn School of Medicine at Mount Sinai, New York, NY, USA.; ^2^ Department of Psychiatry, Icahn School of Medicine at Mount Sinai, NY, New York, USA.; ^3^ BioMedical Engineering and Imaging Institute, Icahn School of Medicine at Mount Sinai, New York, NY, USA.; ^4^ Center for Nutrition and Health Research, National Institute of Public Health, Cuernavaca, Mexico.; ^5^ National Institute of Perinatology, Mexico City, Mexico.; ^6^ National Research Center in Imaging and Medical Instrumentation, Universidad Autónoma Metropolitana, Mexico City, Mexico.

## Abstract

Exposure to neurotoxic metals early in life can disrupt brain development and increase risk of later mental health problems, but vulnerable periods and underlying mechanisms remain unclear. We examined how early life exposures to mixtures of metals affect children’s brain and behavior using naturally shed “baby” teeth as a biomarker of direct exposure. We studied 489 children aged 8 to 14 years and reconstructed weekly concentrations of nine metals from 20 weeks before birth to 40 weeks after birth. We assessed behavior using standardized questionnaires and measured brain structure and function with magnetic resonance imaging. Using lagged weighted quantile sum regression, we identified sensitive developmental windows during which higher exposure to metal mixtures was linked to more behavioral problems, smaller brain volume, reduced brain global efficiency, and weaker white matter integrity. Findings suggest that the developing brain is especially vulnerable to metals in early life, with lasting effects into adolescence.

## Teaser

Baby teeth reveal that early-life exposure to metal mixtures disrupts brain development and increases behavioral problems.

## INTRODUCTION

Throughout development, the brain undergoes a complex series of dynamic processes in which a handful of cells transform into a highly interconnected brain (*1*). Early-life exposures to toxic chemicals, such as neurotoxic metals, can disrupt this process, increasing the risk of mental health issues later in life (*2*–*4*). These early life exposures coincide with sensitive developmental periods during which a child’s brain is particularly susceptible to environmental exposures, known as critical windows of vulnerability. How do early-life metal exposures influence a child’s opportunity for a healthy life? A better understanding of these vulnerable developmental periods, as well as the biological mechanisms at play, would help guide prevention strategies and improve children’s mental health outcomes.

Mental health problems such as depression, anxiety, and attention deficit hyperactivity disorder are increasingly prevalent among children and adolescents, representing a substantial global public health concern. Approximately one in seven young adults worldwide is affected, with 35% of these disorders manifesting by age 14 (*5*). While genetic factors play a role, strong evidence indicates that susceptibility to these conditions may be programmed by events occurring during fetal and early postnatal life, including exposure to neurotoxic metals. One major challenge in investigating these effects (*6*–*9*) is that traditional biomarkers cannot directly measure fetal exposure or provide temporally resolved, retrospective data (*10*). To overcome this, our team has developed and validated the use of deciduous “baby” teeth as a previously unexplored biomarker to measure children’s direct exposure to metals. The growth pattern of these teeth, which begin developing in the second trimester and are naturally shed in childhood, allows us to reconstruct exposure profiles to multiple metals throughout early life on a weekly scale, starting from the second trimester. Prior studies have validated this approach by showing that dentine metal levels correspond to maternal, cord, and infant biomarkers (*11*, *12*) and can be linked to neurodevelopmental outcomes (*2*, *3*, *7*, *13*). This innovative approach offers a unique opportunity to identify highly time-resolved critical windows of developmental susceptibility to metal mixtures that may affect neurodevelopmental outcomes in adolescence.

In this study, we investigated behavioral and magnetic resonance imaging (MRI) phenotypes to capture changes in the brain that may be associated with early-life exposure to metals. We focus on three well-established global MRI phenotypes to capture overall changes in the brain that may be associated with early-life exposure to metals: (i) total brain volume, (ii) global functional network efficiency, and (iii) white matter (WM) integrity. These global MRI phenotypes develop throughout early life, with a maximum growth rate (velocity) occurring during the late fetal, neonatal, and infancy stages (*14*–*16*), and peak in late childhood and adolescence (*14*). Alterations in global MRI phenotypes are known to correlate with maladaptive behavioral outcomes during adolescence. Specifically, reductions in brain volume during adolescence have been strongly associated with cognitive deficits and heightened susceptibility to psychiatric disorders (*17*), decreased global functional connectivity has been linked to neuropsychiatric disorders and impairments in executive function and emotional regulation (*18*), and last, decreased white matter integrity has been associated with slower processing speed and difficulties sustaining attention and learning (*19*–*21*). Prior neuroimaging and toxicological studies further suggest that exposure to neurotoxic metals can alter structural and functional brain development, providing a biologically plausible pathway between environmental exposures and later behavioral outcomes (*22*, *23*). By leveraging a retrospective biomarker of early-life metal exposure (i.e., during maximum velocity of development) and measuring brain outcomes during adolescence (i.e., at peak developmental milestones) (*14*) we identify early-life critical windows of susceptibility and capture the neural mechanisms underlying these associations.

## RESULTS

### Critical windows of susceptibility to metal exposure and behavioral problems

**Fig. 1.**
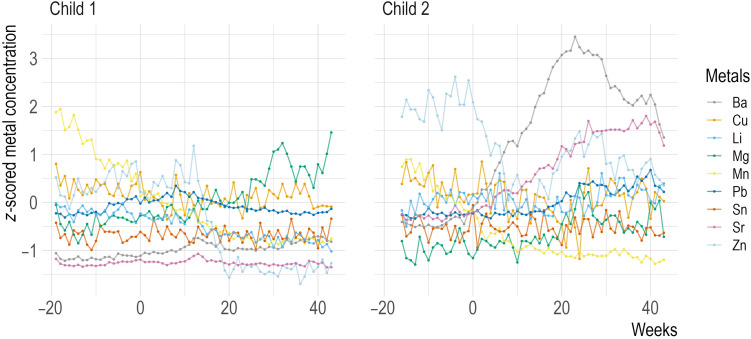
Variability in dentine metal concentrations across development measured in naturally shed deciduous teeth from two study participants. The *y* axis is *z*-scored metal concentrations normalized to Ca. The *x* axis represents weekly concentrations from −20 weeks gestation through 40 weeks postnatal, with “0” representing birth. Metals include Ba, Cu, Li, Mg, Mn, Pb, Sn, Sr, and Zn.

**Fig. 2.**
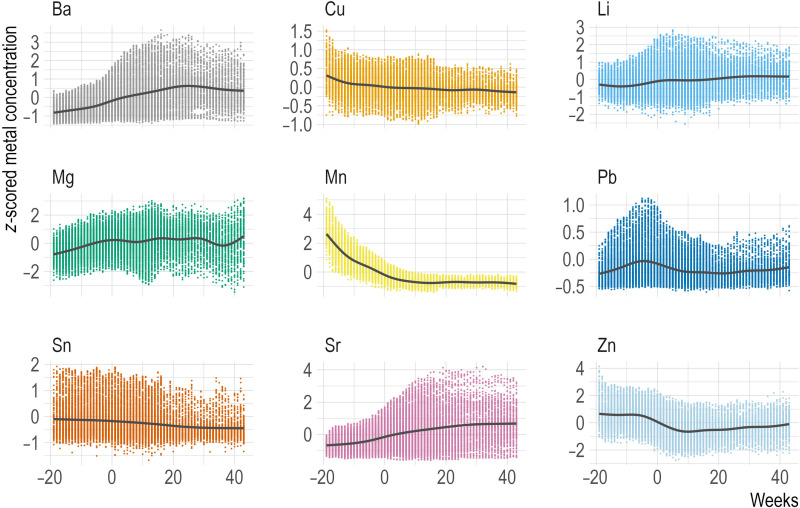
Dentine metal concentrations across development in PROGRESS participants. Colored dots represent individual tooth measurements for 489 participants from PROGRESS with ~60 measurements per participant. Lines represent Loess smoother. The *y* axis is z-scored metal concentrations normalized to Ca. The *x* axis represents weekly concentrations from −20 weeks gestation through 40 weeks postnatal, with 0 representing birth.

To generate high-resolution exposure data capturing the timing and intensity of metal uptake, we collected and analyzed deciduous teeth from 489 children enrolled in the ongoing Programming Research in Obesity, Growth, Environment, and Social Stressors (PROGRESS) study (table S1). Deciduous teeth accumulate metals in an incremental pattern akin to growth rings, encompassing the prenatal and early postnatal periods (*24*). We analyzed the teeth for metals using laser ablation–inductively coupled plasma mass spectrometry (LA-ICPMS) (*25*, *26*). Dentine was sampled along the dentine-enamel junction, and temporal information was assigned after identifying the neonatal line. Approximately 60 sampling points were identified per tooth, spanning from 4 months gestation to 10 months postnatal age, with a sampling frequency of approximately every 7 to 10 days, providing a weekly resolution of nine metals [manganese (Mn), zinc (Zn), lead (Pb), magnesium (Mg), lithium (Li), copper (Cu), strontium (Sr), barium (Ba), and tin (Sn)]. Figure 1 shows temporal maps of the dentine metal mixture containing Ba, Cu, Li, Mg, Mn, Pb, Sn, Sr, and Zn at ~60 time points between the second trimester of pregnancy (4 months gestation) and 10 months of age from two children participating in this study. Figure 2 shows individual concentrations of dentine metals in the full cohort (*n* = 489). Consistent with previous findings (*3*, *23*), dentine Mn levels were highest in the second trimester, declined steeply over the prenatal period, and continued to decline more slowly after birth. Dentine levels of Cu, Li, Mg, Pb, Sn, and Zn were mostly stable over the 14-month sampling period. Dentine Ba, Sr levels demonstrated a slight increase in the first several months after birth.

Behavioral assessments using the *Behavior Assessment System for Children*, second edition (BASC-2), were completed by parents, who rated their child’s behavior. Subjects were between 8 and 12 years old at the time of assessment (mean age: 9.7 years). Our analyses incorporated three composite scores: (i) the behavioral symptoms index (BSI), (ii) internalizing (e.g., anxiety and depression), and (iii) externalizing (e.g., hyperactivity and attention) problems. To test our hypothesis that metal uptake exposure at specific critical windows is associated with behavioral problems, we used lagged weighted quantile sum (L-WQS) regression (*27*, *28*). L-WQS combines the generalized weighted quantile sum (WQS) regression (*29*) algorithm with the distributed lag model (DLM) approach to model time-varying associations between exposure to high-dimensional correlated mixtures and outcomes. A discrete WQS model is applied at each time interval (i.e., weekly), resulting in time-varying estimates of the contribution of each component to construct WQS(t). A reversed DLM is then run relating the outcome variable and time-varying exposures based on WQS indices in association models, adjusted by covariates.

**Fig. 3.**
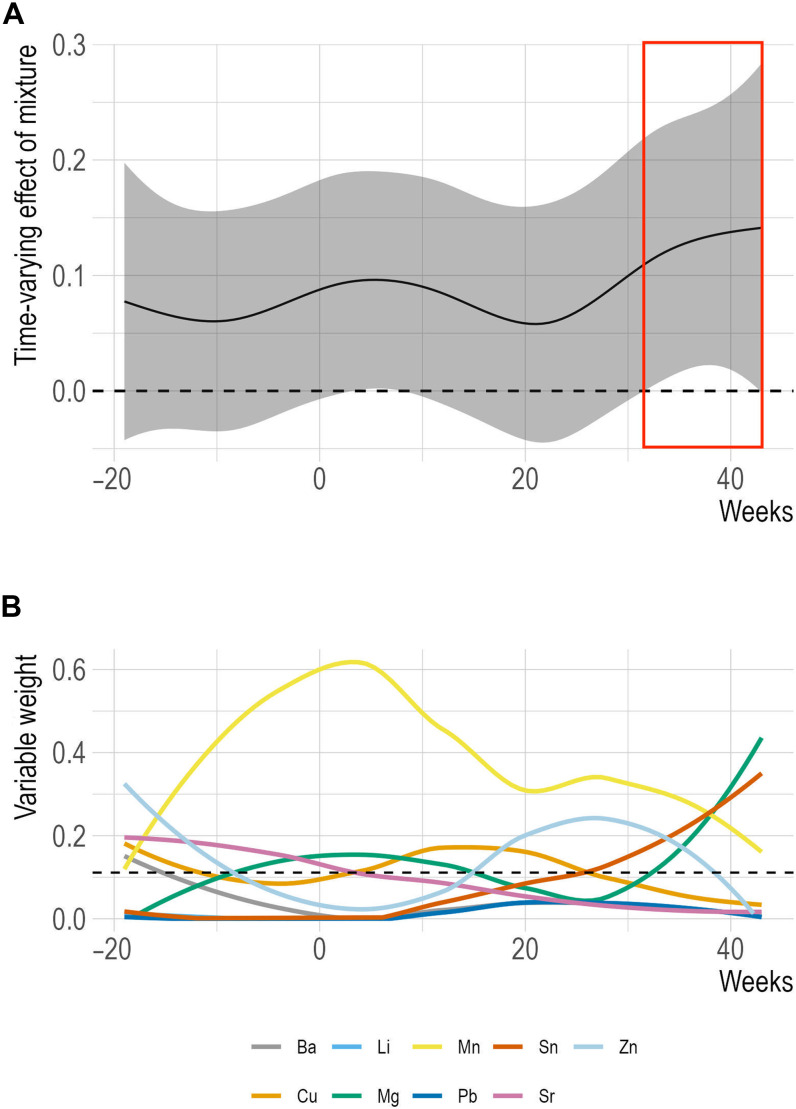
Critical windows of susceptibility to metal exposure and BSI among 395 participants included in the current study. (**A**) L-WQS plots with 95% piecewise confidence intervals demonstrating two critical windows between the metal mixture and BSI (an early postnatal window at weeks 4 to 8 postnatally, maximum β = 0.096 [95% CI: 0.002, 0.19]) and a late postnatal window at weeks 32 to 42 postnatally, maximum β = 0.15 [95% CI: 0.004, 0.28]). The *y* axis represents time-varying association between the metal mixture and BSI. (**B**) Weighted associations of the individual metals driving the observed mixture effect. Weights above the dotted line indicate higher than chance contribution to the mixture effect. In both figures, the *x* axis represents the time since birth, indicating the timing (in weeks) of tooth sampling.

Our results suggest two critical windows of susceptibility in which metal exposure is associated with an increased risk of overall behavioral problems (Fig. 3A): (i) an early postnatal window (weeks 4 to 8 postnatally, maximum β = 0.096 [95% confidence interval (CI): 0.002, 0.19]), and (ii) a late postnatal window (weeks 32 to 42 postnatally, maximum β = 0.15 [95% CI: 0.004, 0.28]). At 5 weeks postnatally (i.e., the week with the strongest association within this first window), a one-quartile increase in the mixture is associated with a 0.1 SD increase in the BSI score. At 38 weeks postnatally (i.e., the week with the strongest association within this second window), a one-quartile increase in the mixture is associated with a 0.13 SD increase in the BSI score. Cumulative effects are reported in table S2. These associations are driven mainly by Mn in the early postnatal period and by Mn, Mg, and Sn in the late postnatal period (Fig. 3B). When internalizing and externalizing problems were examined separately, we did not identify critical windows related to metal exposure (fig. S3).

### Critical windows of susceptibility to metal exposure and global neural phenotypes

To further investigate neurobiological mechanisms underlying the association between early life exposure to metals and behavioral problems, we enrolled a subset of 215 PROGRESS children in an MRI follow-up study between 2019 and 2023. We collected multimodal MRI scans, including T1-weighted anatomical imaging, resting state functional (rs-fMRI), and diffusion tensor imaging (DTI) on a Philips Achieva 3T equipped with an eight-channel head coil at the National Research Center for Medical Instrumentation and Imaging (Ci3M) in Mexico City. T1-weighted anatomical images processing was performed using FreeSurfer analysis suite v6.0.0 (http://surfer.nmr.mgh.harvard.edu/). Processing pipeline included skull stripping; intensity normalization; and voxel segmentation of gray matter (GM), WM, and cerebrospinal fluid (CSF). Labeling of the GM regions was performed using the Desikan-Killiany atlas (*30*) to compute total brain volume. DTI images were reprocessed using FMRIB Software Library v6.0 (FSL) (www.fmrib.ox.ac.uk/fsl). Preprocessing pipeline included eddy-current correction, brain extraction, diffusion tensors fitting, and coregistering fractional anisotropy (FA) images into Montreal Neurological Institute (MNI) brain space. The Johns Hopkins University ICBM-DTI-81 WM labels atlas was used to locate anatomical regions in MNI152 space (*31*), and the fslstats tool was used to calculate the mean values of global fractional anisotropy. rs-fMRI image preprocessing and global efficiency (GE) calculation were performed using SPM12 (*32*), Brain Connectivity Toolbox, and customized scripts, implemented in MATLAB 2016b. Preprocessing pipeline included normalization and coregistration to MNI space, smoothing, and time series extraction. GE was computed using the Brain Connectivity Toolbox.

**Fig. 4.**
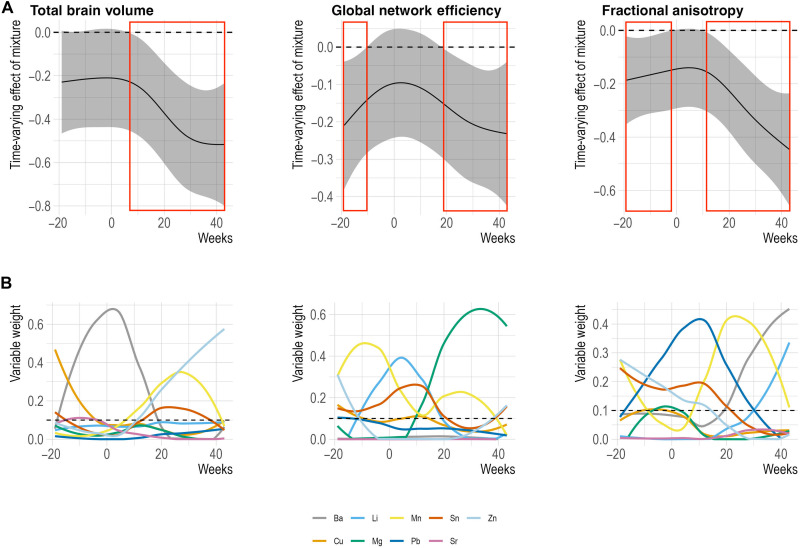
Critical windows of susceptibility to metal exposure and global MRI phenotypes (total brain volume, global network efficiency, and mean FA), among 191 participants included in the current study. (**A**) Lagged WQS plots with 95% piecewise confidence intervals. The *y* axis represents time varying association between joint exposure and MRI phenotypes; total brain volume, global network efficiency, and FA. (**B**) Weighted associations of the individual metals to the observed mixture effects shown in plot A. In all panels, the *x* axis represents the time since birth, indicating the timing (in weeks) of tooth sampling.

We used L-WQS to examine associations between the temporally resolved dentine metal mixture and global MRI phenotypes: (i) total brain volume, (ii) global network efficiency, and (iii) FA. For all global MRI phenotypes, we detect critical windows of susceptibility in which metal exposure is associated with decreased brain volume, functional connectivity, and white matter microstructure integrity. We observed a postnatal critical window spanning weeks 15 to 43 during which the metal mixture was associated with reductions in total brain volume (maximum β = −0.46 [95% CI: −0.68, −0.25]). At 32 weeks postnatally, a one-quartile increase in the mixture is associated with a 0.46 SD decrease in total brain volume (Fig. 4A). The association was driven mainly by Zn, Sn, and Mn (Fig. 4B).

We observed two critical windows spanning weeks −19 to −8 prenatally and weeks 17 to 43 postnatally, during which the metal mixture was associated with reductions in global network efficiency, a measure of functional connectivity (maximum prenatal β = −0.18 [95% CI: −0.34, −0.02] and maximum postnatal β = −0.21 [95% CI: −0.35, −0.06]). At 17 weeks prenatally, a one-quartile increase in the mixture is associated with a 0.18 SD decrease in global network efficiency, and at 33 weeks postnatally, a one-quartile increase in the mixture is associated with a 0.21 SD decrease in global network efficiency (Fig. 4A). The association was driven mainly Mn in the prenatal window and by Mg and Pb in the postnatal window (Fig. 4B).

We observed two critical windows spanning weeks −19 to 0 prenatally and weeks 12 to 43 postnatally, during which the metal mixture was associated with reductions in FA, a measure of white matter integrity (maximum prenatal β = −0.19 [95% CI: −0.33, −0.04] and maximum postnatal β = −0.39 [95% CI: −0.59, −0.19]). At 15 weeks prenatally, a one-quartile increase in the mixture is associated with a 0.19 SD decrease in FA, and at 33 weeks postnatally, a one-quartile increase in the mixture is associated with a 0.32 SD decrease in FA (Fig. 4A). Cumulative effects are reported in table S2. The association was driven mainly Mn, Zn, Cu, and Mg in the prenatal window and by Mn, Ba, and Li in the postnatal window (Fig. 4B).

## DISCUSSION

In this study, we reconstructed temporally resolved concentrations of a nine-metal mixture throughout early life to identify critical windows of developmental susceptibility that may be associated with behavioral and neural phenotypes in adolescence. By considering metals as a changing mixture across gestation and early childhood, we captured the complexity of early life, time-varying metal exposure and its effect on children’s long-term neurodevelopment. Our findings identify multiple critical windows during which metal uptake affects brain and behavior. These windows include early and late postnatal periods linked to behavioral problems (driven mainly by Mn, Mg, and Sn), a postnatal period linked to reduced brain volume (driven mainly by Zn, Sn, and Mn), prenatal and postnatal periods linked to decreased global network efficiency (driven mainly by Mn, Mg, and Pb), and prenatal and postnatal periods linked to reduced white matter integrity (mainly Mn, Zn, Cu, Mg, Ba, and Li). These findings highlight that the effects of metal exposure are influenced by coexposure to neuroactive metals and, crucially, by the timing of the exposure.

Our findings confirm previous research indicating that early life, encompassing both prenatal and postnatal periods, is critical for the development of neural circuits and connections that significantly influence behavioral outcomes in adolescence (*3*, *23*, *33*, *34*). Understanding early life risk factors through a developmental lens is particularly relevant to adolescent behavioral problems, as these issues often originate from early developmental events (*35*, *36*) and cannot be explained by genetic risk alone. While critical windows for metal exposure have been previously identified (*23*, *33*, *37*), this study is the first to capture these windows with high resolution on a weekly scale using our dentine biomarker. When we investigated subscores for internalizing and externalizing problems separately, we did not identify significant associations. These results may be explained by sex/gender-specific effects operating in opposite directions in females and males. Specifically, females tend to exhibit higher levels of internalizing problems, whereas males tend to exhibit higher levels of externalizing problems (*38*). Consequently, the overall effect of metal exposure on behavior might be masked when examining the entire population, as these opposing effects may cancel each other out. While our study is underpowered to examine sex differences, further research is needed to explore these effects in more detail.

Across our results, the postnatal period of 6 to 9 months consistently emerges as a critical window. These results are biologically plausible as this time frame is marked by significant changes in infants’ nutrition and lifestyle, as well as heightened neurodevelopmental plasticity (*39*–*41*). During this period, infants typically transition from exclusive breastfeeding or formula feeding to the introduction of solid foods. This dietary shift may influence the absorption and metabolism of nutrients and toxins, potentially increasing susceptibility to metals (*42*, *43*). In addition, infants begin to crawl and interact more with their environment, possibly elevating exposure through increased ingestion or inhalation of metals (*44*, *45*). Furthermore, the maturation of the blood-brain barrier during this stage may affect how toxins, including metals, are processed and their potential impact on the developing brain. While the blood-brain barrier becomes more selective, it is still not fully mature, allowing certain neurotoxic substances to penetrate more easily than in later stages of life (*46*). These changes coincide with a period of rapid brain growth and synaptic pruning (*47*). Our study highlights that exposure to toxic metals during the postnatal window of 6 to 9 months can interfere with these developmental processes, leading to detectable alterations in brain volume, functional connectivity, and white matter integrity later in life. These results suggest that interventions during this period could be crucial in promoting mental health outcomes in adolescence and in mitigating adverse effects on brain development.

When examining the metals that contribute most to these associations, Mn appears to play a critical role. This finding aligns with previous studies indicating that Mn exposure during specific critical windows of brain development can disrupt normal development (*9*, *10*, *23*). Infants can be exposed to Mn through various routes, including inhalation, dietary intake, drinking contaminated water (*48*), and living in proximity to ferroalloy industries (*49*). High levels of Mn have been shown to alter neuron function (*50*), increase oxidative stress (*51*), accumulate in the basal ganglia (*52*), and affect cognition and behavior (*53*, *54*). Results from these prior studies provide some insight into the timing of Mn exposure and the developmental windows of susceptibility but are unable to provide temporal exposure information available through dentine biomarkers and do not account for coexposure to other neuroactive metals. Our results suggest that the impact of early life Mn exposure may depend both on the timing and the dose of the exposure and underscores the importance of regulation and policies to reduce Mn exposure. Beyond Mn, our results identify Mg and Zn as important contributors to the overall mixture effect. Convergent human biomarker studies implicate these metals in adverse neurodevelopmental outcomes, including autism spectrum disorder, attention-deficit/hyperactivity disorder, and behavior problems in school-age children (*3*, *13*, *55*, *56*). Given these metals’ roles in *N*-methyl-D-aspartate receptor regulation and synaptic signaling, these data provide biological plausibility for the time-specific Mg and Zn contributions we observe (*57*–*59*). Although Pb is a well-established neurotoxicant, we did not observe major contributions of Pb exposure to the mixture effect. One possibility is that Pb-related effects are more pronounced for cognitive or academic outcomes, which were not assessed here, and may be less readily captured by the BASC-2 behavioral indices.

To our knowledge, this is the first study to examine time-resolved associations between coexposure to a mixture of metals and both brain and behavior outcomes in adolescence. We acknowledge several limitations. While our study provides insights into the neural mechanisms underlying the observed associations between metal exposure and behavioral problems, our sample size did not allow for a direct test of brain-behavior associations, as reproducible associations would require thousands of individuals (*60*). In addition, although sex-specific effects have been well-documented in associations between metal exposure and neuroimaging outcomes (*23*), our sample size limited the statistical power to test sex-specific effects. Participants were drawn largely from low-income communities in Mexico City, resulting in limited variability in socioeconomic measures such as parental education or income; therefore, these covariates were not included in our models and generalizability beyond this Mexico City cohort may be limited. While our use of deciduous teeth provides retrospective data on early life exposures, deciduous teeth begin developing only in the early second trimester, and we cannot examine the effects of very early metal exposure on neurodevelopmental outcomes occurring in the first trimester. While our primary analyses constrained the mixture effect in the adverse direction, it is important to acknowledge that several of the metals driving our associations—such as Zn, Mn, and Mg—are essential nutrients, and prior work suggests that both deficiency and excess may be harmful. Finally, while the lagged WQS method provides a comprehensive profile of the time-varying metal mixture, it does not allow for estimating potentially multiplicative effects (i.e., metal interactions).

In this study, we identified critical windows of susceptibility to metal mixture exposure, affecting both brain and behavior. Our findings indicate that the developing brain is particularly vulnerable to metal exposure during early life, with a significant impact observed during the 6 to 9 months period, persisting through adolescence. This research revolutionizes our understanding of how the timing and concentration of metal exposure influence brain development and behavior, offering mechanistic insights into metal neurotoxicity. It underscores the importance of detecting precise exposure windows to assess the risks and effects of metals on neural development accurately. Further research aimed at identifying sensitive developmental critical windows to environmental insults will improve public health strategies. Future research should focus on linking sex-specific neural correlates with behavioral and neural outcomes.

## MATERIALS AND METHODS

### Study participants

This study includes children enrolled in the ongoing longitudinal birth cohort study PROGRESS (Programming Research in Obesity, Growth, Environment, and Social Stressors) based in Mexico City. The PROGRESS study has been described in detail elsewhere (*61*). Briefly, between 2007 and 2011, pregnant women attending prenatal consultations at four clinics affiliated with the Mexican Social Security System in Mexico City were approached for potential enrollment. Eligibility criteria included: being <20 weeks pregnant, age ≥ 18 years (Mexican legal voting age), the absence of heart or kidney diseases, the possession of a telephone, intention to reside in Mexico City for the subsequent 3 years, no regular use of steroids or anti-epilepsy drugs, and no daily alcohol consumption. We actively followed 760 mother-child dyads at the National Institute of Perinatology in Mexico City, conducting longitudinal assessments. At the 9-year visit, behavioral assessments using the BASC-2 were administered on the basis of parent reports. From the enrolled children in PROGRESS, between 2019 and 2023, a subset of 215 children completed multimodal MRI scans including T1-weighted anatomical imaging, resting state functional (rs-fMRI), and DTI. An experienced research coordinator from INSP contacted eligible participants for verbal consent. Written informed consent was obtained from all participants at the beginning of the study visit. The study received approval from the internal review boards of the Icahn School of Medicine at Mount Sinai (STUDY-12-00751D) and the National Institute of Public Health in Mexico (CI-1614-29052019).

### Exposure assessment: Dentine biomarkers

Deciduous teeth devoid of noticeable defects such as caries and extensive wear were analyzed for metals using LA-ICPMS. Methods and validation procedures have been described in detail elsewhere (*25*, *26*). Briefly, deciduous teeth accumulate metals in an incremental pattern akin to growth rings, encompassing the prenatal and early postnatal periods (*24*). Dentine was sampled along the dentine-enamel junction and temporal information assigned after identifying the neonatal line (a histological feature present in enamel and dentine at birth) and daily growth incremental markings. Approximately 60 sampling points were identified per tooth, spanning from 4 months gestation to 10 months postnatal age, with a sampling frequency of approximately every 7 to 10 days, providing a weekly resolution of metal uptake. Weekly exposure to nine metals (Mn, Zn, Pb, Mg, Li, Cu, Sr, Ba, and Sn) was estimated. Metal intensities at each sampling point were normalized to calcium (Ca) and corrected to external standard NIST610 to account for variations in mineral density between samples and instrumental drift during analysis. Values are reported as a ratio of metal to Ca.

### Behavioral assessments

The BASC-2 (*62*) was administered to parents of subjects to assess their child’s behavior when children were aged 8 to 11 years. The BASC-2 is a reliable parent-report assessment tool for children’s behavior and self-perceptions, exhibiting high test-retest reliability (*r* = 0.84) and internal consistency. Trained psychologists administered the BASC-2 in Mexican-Spanish using the translated text provided by the publisher (Pearson Clinical). Our analyses incorporated three composite scores: (i) the BSI, (ii) internalizing (e.g., anxiety and depression), and (iii) externalizing (e.g., hyperactivity and attention) problems. We used for all scales the *T* score indicating the distance from the norm mean. The BASC-2 mean *T* score is 50, with an SD of 10. *T* scores higher than 70 are considered in the clinical range. Of the 395 participants who completed the BASC, 15 (3.8%) scored in the clinical range.

### MRI data acquisition

MRI scans including T1W-3D, rs-fMRI, and DTI were acquired on a Philips Achieva 3T equipped with an eight-channel head coil (Sense Head 8) at the National Research Center of Medical Instrumentation and Imaging (Ci3M) in Mexico City. The T1W-3D-TFE sequence had the following parameters: repetition time/echo time (TR/TE) = 7.5 ms/3.5 ms; field of view (FOV) = 25 cm by 25 cm; matrix = 228 by 228; slice thickness = 1.2 mm. The DTI Pulsed-Gradient Spin Echo sequence had the following parameters: 32 directions; TR/TE = 3525 ms/92 ms; FOV = 22.4 cm by 22.4 cm; matrix = 128 by 128; slice thickness = 2 mm; *b* value = 1200 s/mm^2^. The 10-min resting-state fMRI field–echo–echo planar imaging (EPI) sequence had the following parameters: TR/TE = 2000 ms/27 ms; FOV = 22 cm by 22 cm; matrix = 80 by 80; slice thickness = 3 mm. During the rs-fMRI acquisition, lights were turned off, and subjects were instructed to keep their eyes open watching an inscapes video, to not think of anything specific, and not to fall asleep.

### Neuroimaging data preprocessing

#### Structural MRI scans

Image processing was performed using FreeSurfer analysis suite v6.0.0 (http://surfer.nmr.mgh.harvard.edu/). All images were processed individually using FreeSurfer. FreeSurfer processing steps have been described in detail previously (*63*). Briefly, the analysis stream includes converting raw DICOM data to “MGZ files”; skull stripping; intensity normalization; and voxel segmentation of GM, WM, and CSF. The labeling of the GM regions was performed using the Desikan-Killiany atlas (*30*).

#### Diffusion-tensor imaging

DTI were reprocessed using FMRIB Software Library v6.0 (FSL) (www.fmrib.ox.ac.uk/fsl). First, images were eddy current corrected using eddy correct from the FMRIB’s Diffusion Toolbox (FDT) diffusion toolbox (version 3.0). Next, we performed brain extraction using Brain Extraction Tool (BET) (threshold of 0.3) and fitted diffusion tensors using DTIFIT from the FDT diffusion toolbox, creating single FA images for each subject. Coregistered FA images in MNI brain space were computed using the Tract-Based Spatial Statistics (TBSS) (*64*) workflow. The procedure involves skeletonization of the FA images to obtain centers of white matter tracts, thresholding (FA > 0.2) of the FA skeletonized image to suppress areas of low mean FA and/or high intersubject variability, and projecting each subject’s FA image onto the skeleton. The Johns Hopkins University ICBM-DTI-81 WM labels atlas was used to locate anatomical regions in MNI152 space (*31*). Mean FA was calculated for each atlas-defined region on each participant’s coregistered MNI FA image computed from TBSS. The fslstats tool was used to calculate the mean values of global FA.

#### Resting state–fMRI

Image preprocessing and GE calculation were performed using SPM12 (*32*), Brain Connectivity Toolbox (*65*), and customized scripts, implemented in MATLAB 2016b (The MathWorks Inc., Natick, Massachusetts). Image preprocessing: For each subject, the structural magnetic resonance image was coregistered and normalized against the MNI template and segmented to obtain WM, GM and, CSF probability maps in the MNI space. FMRI data were spatially realigned, coregistered to the MNI-152 EPI template, and subsequently normalized using the segmentation option for EPI images in SPM12. All normalized data were denoised using Independent Component Analysis-Automatic Removal of Motion Artifacts (ICA-AROMA) (*66*). In addition, spatial smoothing was applied (8 mm) to the fMRI data. No global signal regression was applied. On the basis of the Harvard-Oxford atlas (*67*), 111 regions of interest (ROI; 48 left and 48 right cortical areas; 7 left and 7 right subcortical regions and 1 brainstem) were defined. The T1-weighted images were segmented and affine-registered to MNI152 space using FLIRT (FSL), and the transformations were then applied to the individual brain areas’ labels. For each ROI, a time series was extracted by averaging across voxels per time point. To facilitate statistical inference, data were “prewhitened” by removing the estimated autocorrelation structure in a two-step generalized linear model procedure (*68*). In the first step, the raw data were filtered against the six motion parameters (three translations and three rotations). Using the resulting residuals, the autocorrelation structures present in the data were estimated using an autoregressive model of order 1 (*69*) and then removed from the raw data. Next, the realignment parameters, WM and CSF signals, were removed as confounders on the whitened data. Network analysis: GE was computed using the Brain Connectivity Toolbox (*65*) on the defined ROI time course data per subject. GE builds on the concept of efficient integration of communication in a network at whole level. It is defined as the inverse of the average characteristic path length between all nodes in the networks (*70*, *71*). For each individual node, with each node defined as an ROI, the shortest number of steps required to traverse a path from one node to another was computed. Then, the average number of shortest steps to all defined nodes was computed separately for each node. To correct for the total number of connections between nodes, the inverse of the average number of shortest steps for each node was summed across all network nodes and normalized. GE is a scaled measure ranging from 0 to 1, with a value of 1 indicating maximum GE in the brain.

#### Statistical analyses

We conducted standard univariate data explorations and examined the distributional plots of metal concentrations measured between 4-month gestational age (−20 weeks) to 10 months postnatally (44 weeks), BASC-2 scores, and neural phenotypes. Appropriate transformations (e.g., *z* score) were performed, as necessary, to satisfy model assumptions. Each metal’s concentration was normalized to the value of calcium and *z*-scored, constructing a relative concentration value at each time point. Exposure outliers were identified using the interquartile range (IQR) per each sampling time point and for each metal, i.e., a data point was considered an outlier if it was over 1.5 times the IQR below the first quartile or 1.5 times the IQR above the third quartile of each metal and each week. Outliers were flagged for visualization purposes only and were retained in all statistical analyses.

To test our hypothesis that metal exposure at specific critical windows is associated with behavioral problems and neural phenotypes, we used L-WQS regression (*27*, *28*) with a random subset (*72*). L-WQS combines the generalized WQS regression (*29*) algorithm with the DLM approach (*73*) to model time-varying associations between exposure to high dimensional correlated mixtures and outcomes. The L-WQS approach has been described in detail in (*28*). Briefly, a discrete WQS model is applied at each time interval (i.e., weekly), resulting in time-varying estimates of the contribution of each component to construct WQS(*t*). The L-WQS then takes the time-varying mixture index as inputs to a reverse DLM, i.e., WQS(*t*) = β_0_(*t*) + β_1_(*t*)*Y* + γ*z* + *u* + ε, which estimates the significance of associations between the weighted index and outcome. Confidence intervals for β_1_(*t*) are constructed in the final step. The lag structure was modeled using penalized cubic regression splines (bs = “cr”) within a generalized additive mixed model framework, using a basis dimension of *k* = 5 to 7. Knots were placed evenly across the range of weekly exposure values, and spline smoothness was controlled using the standard second-order difference penalty, with smoothing parameters estimated via restricted maximum likelihood, allowing flexible, smooth estimation of time-varying associations. A sensitivity analysis varying the basis dimension showed stable results across specifications (fig. S3).

When the estimate of β_1_(*t*) is positive and significant, the mixture is positively correlated with the outcome, and vice versa. Estimated wj(*t*) reveals the components contributing to the mixture effect. Models were estimated across 1000 bootstrap samples. Metal concentrations were ranked in quartiles. For all analyses, the directionality of the association of the WQS index was constrained in the direction of the hypothesized harmful effects of the metal mixture, i.e., positive direction when modeling behavioral problems and negative direction when modeling MRI phenotypes. All L-WQS models were adjusted for age and sex. L-WQS analyses were implemented in R (v4.2.1) using the lwqs and gamm4 packages.
