# Supplementary material for: Fetal and postnatal metal metabolism–related changes in brain function are associated with childhood behavioral deficits
Source: Sci Adv. 2026 Apr 24;12(17):eadz1340. doi: 10.1126/sciadv.adz1340 (PMC13108532; doi:10.1126/sciadv.adz1340)
Supplement: Supplementary file 1 — Figs. S1 to S3 Tables S1 and S2 [file sciadv.adz1340_sm.pdf]

Science Advances
AAAS

Supplementary Materials for

Fetal and postnatal metal metabolism–related changes in brain function are associated with childhood behavioral deficits

Elza Rechtman *et al.*

Corresponding author: Manish Arora, manish.arora@mssm.edu

Sci. Adv. **12**, eadz1340 (2026)
DOI: 10.1126/sciadv.adz1340

This PDF file includes:

Figs. S1 to S3
Tables S1 and S2

Supplemental Figure 1. Time-varying correlations among dentine metals. Pairwise Pearson correlations (r) among nine dentine metals (Mn, Zn, Mg, Cu, Sn, Sr, Li, Ba, Pb) at approximately 60 time points between the second trimester of pregnancy (4 months gestation) and 10 months of age from two children participating in this study. Colors encode correlation strength (blue = positive, red = negative, white ≈ 0); diagonal cells reflect r = 1.

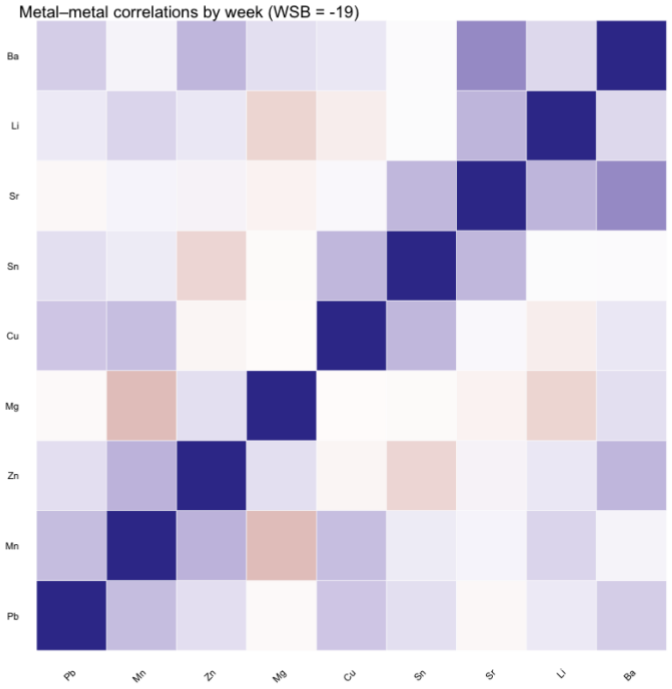

Supplemental Figure 2. Results from L-WQS analyses of metal exposure and BASC-2 externalizing and internalizing problems among 395 participants included in the current study.

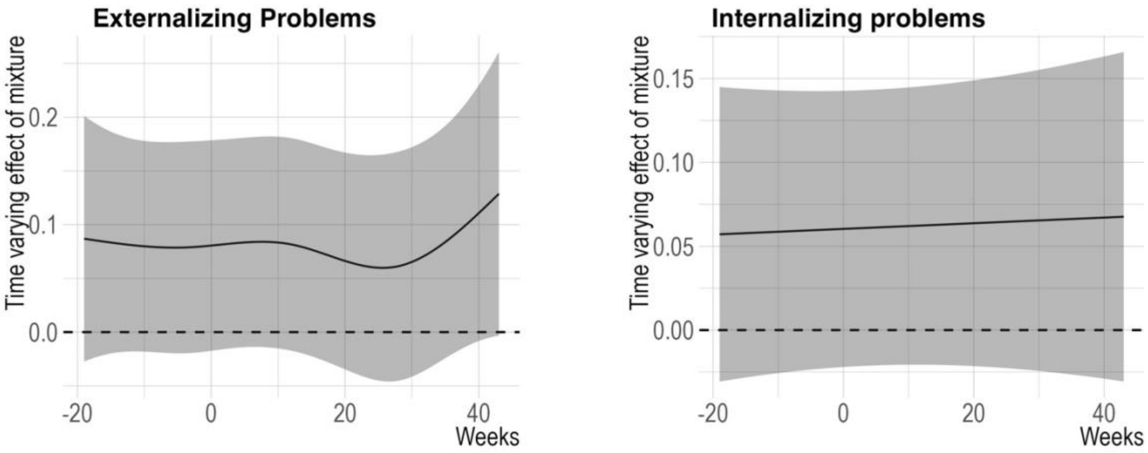

L-WQS plots with 95% piecewise confidence intervals demonstrate no critical windows between the metal mixture and externalizing and internalizing problems. The Y axis represents time-varying correlation between the metal mixture and outcome.

Supplemental Figure 3. **Sensitivity of Distributed Lag Model (DLM) Estimates to Spline Basis Dimension.** Time-varying mixture effects from DLMs estimated using penalized cubic regression splines with alternative basis dimensions (k = 4, 5, 6, 7). The overall timing, and relative magnitude of critical windows remained consistent across spline specifications, demonstrating results were stable with respect to the choice of basis dimension. All models used evenly spaced knots and smoothing penalties estimated via restricted maximum likelihood (REML).

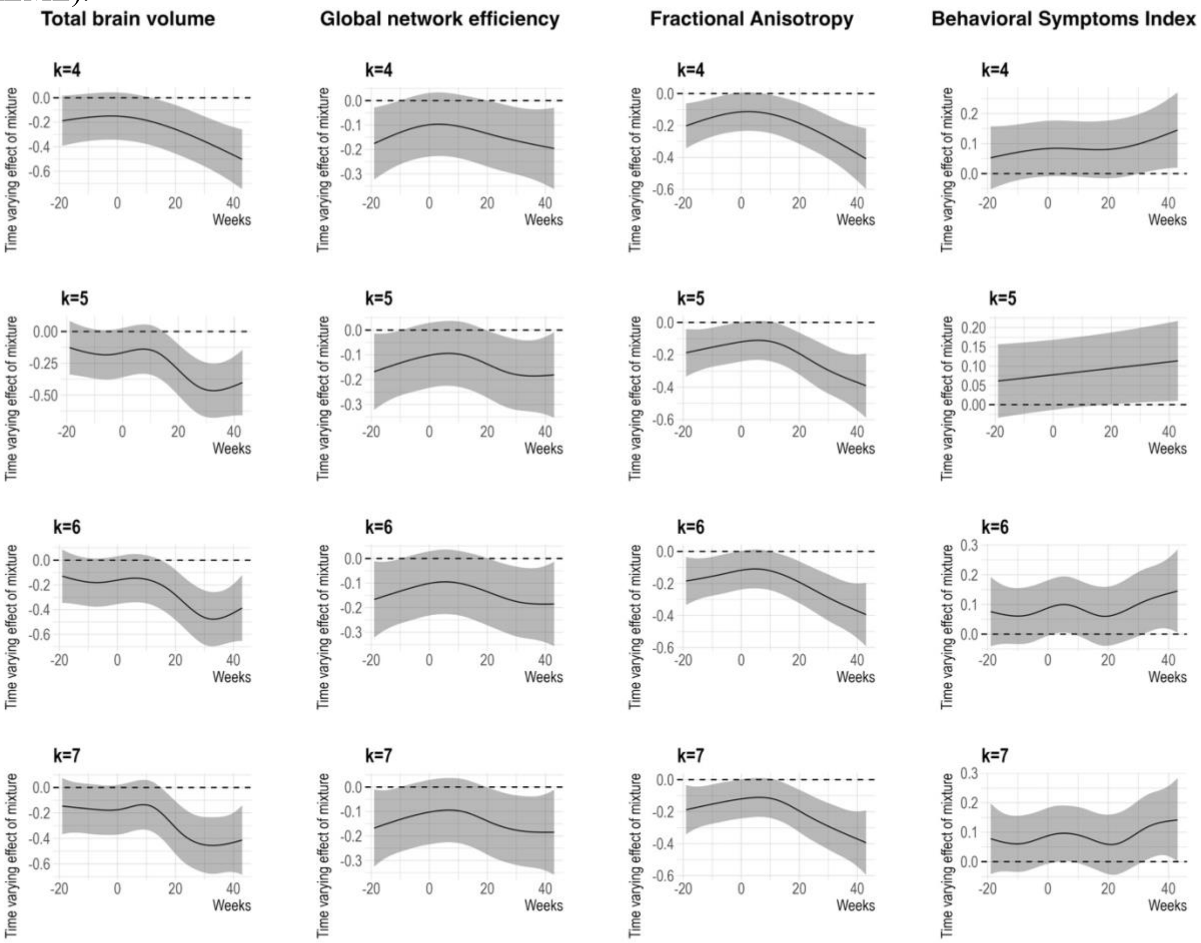

Supplemental Table 1. Sociodemographic characteristics of 489 participants from the PROGRESS study.

	Overall (N=489)
**Sex**	
Male	255 (52.1%)
Female	234 (47.9%)
**Age at BASC (n = 395)**	
Mean (SD)	9.71 (0.679)
Median [Min, Max]	9.69 [8.08, 12.1]
**Age at scan (n = 191)**	
Mean (SD)	12.7 (1.42)
Median [Min, Max]	13.1 [8.38, 14.8]
**Behavioral Symptoms Index**	
Mean (SD)	52.3 (9.82)
Median [Min, Max]	51.0 [34.0, 89.0]
**Externalizing Problems**	
Mean (SD)	50.9 (8.74)
Median [Min, Max]	49.0 [34.0, 82.0]
**Internalizing Problems**	
Mean (SD)	53.6 (10.5)
Median [Min, Max]	52.0 [33.0, 101]

Note. BASC: Behavior Assessment System for Children, max: maximum; min: minimum.

Supplemental Table 2. **Cumulative β across critical windows of susceptibility to metal exposure and all outcomes: Behavioral Symptoms Index (BSI), total brain volume, global network efficiency, and global fractional anisotropy.**

Outcome	Critical window	Maximum β	Cumulative β across the critical window
Behavioral Symptoms Index (BSI)	weeks 4 to 8 postnatally	0.96 [95% CI 0.002, 0.19]	0.67
Behavioral Symptoms Index (BSI)	weeks 32 to 42 postnatally	0.15 [95% CI 0.004, 0.28]	2.08
Total brain volume	weeks 15 to 43 postnatally	-0.46 [95% CI -0.68, -0.25]	-20.45
Global network efficiency	weeks -19 to -8 prenatally	-0.18 [95% CI -0.34, -0.02]	-3.01
Global network efficiency	weeks 17 to 43 postnatally	β = -0.21 [95% CI -0.35, -0.06]	-8.78
Global Fractional Anisotropy	weeks -19 to 0 prenatally	-0.19 [95% CI -0.33, -0.04]	-5.13
Global Fractional Anisotropy	weeks 12 to 43 postnatally	-0.39 [95% CI -0.59, -0.19]	-15.31

Note. Cumulative β is the sum of weekly β(t) over the window (Δt = 1 week), i.e., the expected change in the outcome for a one-quartile increase in the mixture sustained across the window. Weeks are relative to birth (− prenatal, + postnatal); models adjust for child age at outcome and sex, with effect direction constrained toward adverse associations.
